# Ocular Surface Microbiota and Corneal Transplant Outcome: Is There a Link?

**DOI:** 10.3390/biomedicines13040972

**Published:** 2025-04-16

**Authors:** Michele Potenza, Antonio Moramarco, Annalisa Astolfi, Carmen Ciavarella, Luigi Fontana, Piera Versura

**Affiliations:** 1 Ophthalmology Unit, DIMEC, Alma Mater Studiorum University of Bologna, 40138 Bologna, Italy; michele.potenza2@studio.unibo.it (M.P.); luigi.fontana6@unibo.it (L.F.); 2 IRCCS Azienda Ospedaliero Universitaria di Bologna, 40138 Bologna, Italy; antonio.moramarco@aosp.bo.it (A.M.); annalisa.astolfi@unibo.it (A.A.); 3 DIMEC, Alma Mater Studiorum University of Bologna, 40138 Bologna, Italy; carmen.ciavarella2@unibo.it

**Keywords:** ocular surface microbiota, corneal transplantation, graft rejection, microbial dysbiosis, immune modulation

## Abstract

Recent research has highlighted the critical role of microbiota in organ transplant outcomes, particularly in the gut. However, the impact of ocular surface microbiota (OSM) on corneal transplantation remains largely unexplored. This piece examines the potential connection between OSM imbalances and corneal graftoutcomes, suggesting that microbial shifts could influence immune responses and transplant success. The OSM, though characterized by low microbial density, plays a critical role in local immune modulation and ocular surface homeostasis. Dysbiosis in this microbiota may compromise the immune privilege of the cornea, potentially increasing the risk of graft rejection. Looking at gut microbiota studies, where dysbiosis has been linked to graft failure, it is reasonable to hypothesize that similar mechanisms might be at play on the ocular surface. Disruptions in cornea’s immune tolerance pathways, such as anterior chamber-associated immune deviation (ACAID), may lead to pro-inflammatory responses that threaten graft survival. In addition, ocular surface diseases such as dry eye disease, microbial keratitis, and allergic conjunctivitis, already associated with OSM dysbiosis, may further exacerbate post-transplant complications. Despite the lack of direct studies linking OSM to corneal transplant outcomes, this opinion piece highlights the necessity for future research. Standardizing microbiota analysis methodologies and exploring therapeutic interventions, such as ocular probiotics, could open new roads for improving corneal transplant success and patient prognosis.

## 1. Introduction

Significant advances have been made in understanding the role of microbiota in organ transplantation, particularly in relation to gut microbiota. However, research exploring the ocular surface microbiota (OSM) in corneal transplantation remains limited. While intestinal dysbiosis has been well-documented in transplant outcomes—affecting everything from immune response to graft rejection—the potential influence of microbial imbalances on the ocular surface is still unclear. This opinion piece aims to elucidate the role of OSM in corneal transplantation, hypothesizing its potential impact on immune modulation and post-transplant outcomes. If a relationship between microbiota alterations and negative transplant outcomes is established, this canopen new therapeutic avenues for restoring or maintaining a healthy ocular surface microbiota to optimize graft success and improve clinical prognosis.

## 2. The Ocular Surface Microbiota: Composition and Function

The OSM comprises the community of microorganisms residing on the surface of the eye, including the conjunctiva, cornea, eyelids, and tears. Despite its low bacterial density, the OSM plays a critical role in maintaining the health and physiological balance of the ocular surface by contributing to local immunity and protecting against pathogenic invasion [1,2]. It helps modulate the immune response, distinguishing between commensal and pathogenic organisms, thereby supporting the ocular surface’s defense mechanisms, including the tear film and mucosal immunity [3].

The healthy OSM is dominated by bacteria from three major phyla: Proteobacteria, Firmicutes, and Actinobacteria. At the genus level, the most common genera are *Propionibacterium*, *Corynebacterium*, *Staphylococcus*, and *Streptococcus*. However, the exact composition of the OSN varies among individuals and can be influenced by several factors, including age, sex, health status, contact lens use, and environmental conditions, and at present no “core” member of OSN has been established at the species level [4,5].

Metataxonomic studies have revealed a remarkable bacterial diversity in the OSM, with over 100 species identified per individual and potentially greater variety compared to the gut [5].

Limited information is available regarding the presence of fungi and viruses in healthy eyes. Fungi are less common than bacteria in the OSM but may still be found in healthy individuals [6,7,8]. As for viruses, a study identified a resident DNA virome in the healthy conjunctiva, characterized by low abundance of viral species [9].

## 3. Methodologies for OSM Identification and Quantification

Traditionally, studies on the OSN relied on culture-based methods. However, these approaches were limited, as only a small proportion of ocular bacteria could be successfully cultured in the laboratory. The advent of next-generation sequencing (NGS) has revolutionized the study of microbiota, allowing for more comprehensive and accurate characterization of its diversity. Despite this progress, the standardization of sequencing methodologies remains an ongoing challenge [10].

Among these methods, the 16S rRNA gene sequencing has been the most widely used technique in microbiome research to date. For low-abundance microbiomes, this method has the advantage of selectively amplifying the input material via PCR prior to sequencing, specifically targeting the bacterial 16S rRNA gene. However, this amplification step introduces biases influenced by the initial template concentration, DNA extraction method, and the number of PCR cycles used. Additionally, 16S rRNA gene sequencing is limited to bacteria, omitting other domains of life, which represents another drawback [11].

Whole-metagenome shotgun sequencing is an emerging sequencing technology, which provides a more comprehensive view of taxonomic composition and improves the study of functional profiles within microbiomes as compared to 16S rRNA gene sequencing. This approach involves the parallel sequencing of fragmented DNA from a sample, followed by assembly into longer reads that are mapped to a reference sequence database [12].

One of the key advantages of shotgun sequencing is that it does not require targeted gene amplification, although global amplification can be included for samples with low DNA content. Its primary advantage lies in its ability to sequence DNA regardless of its origin, thereby detecting viruses, archaea, and eukaryotes in addition to bacteria. Furthermore, shotgun sequencing increases taxonomic resolution and provides deeper genomic insights [13].

## 4. Microbial Diversity: A Key Indicator of OSM Health

The concept of diversity is fundamental to understanding the complexity and health of a microbial ecosystem, such as the OSN, and it refers to the variety of species present in each environment and their relative distribution. A diverse microbial community is generally considered healthier and more resilient, being better able to withstand disturbances and perform a broader range of beneficial functions for the host. The purpose of this manuscript does not allow for a detailed technical analysis, but a general overview of the most frequently calculated metrics can be informative and beneficial for readers [14].

Alpha diversity (α-diversity) measures diversity within a single sample and includes the following aspects: richness, referring to the number of distinct species present in the sample, and evenness, referring to the distribution of species in terms of abundance. A community with high evenness has species present in similar proportions, while a low-evenness community is dominated by a few species.

Combined indices: metrics such as the Shannon Index and the Inverse Simpson Index incorporate both richness and evenness to provide a more comprehensive measure of α-diversity.

Beta diversity (β-diversity) assesses differences in community composition or structure between two samples. For instance, β-diversity can compare the microbiota of different individuals or the microbiota of the same individual at different time points. UniFrac distance assessmentis a robust method for comparing microbial community differences (β-diversity), measuring the proportion of branch lengths shared between samples on a phylogenetic tree. Principal Coordinate Analysis (PCoA) is commonly used to visualize UniFrac distances between samples in two- or three-dimensional space.

Gamma diversity (γ-diversity) measures the overall diversity of the entire ecosystem under consideration. For example, γ-diversity might refer to the diversity of the OSN within an entire population.

The literature emphasizes that various diversity indices provide complementary insights into the quality of a microbial community. For instance, a decrease in α-diversity may indicate the loss of beneficial species or an overgrowth of potentially pathogenic species [15].

## 5. Lessons from the Gut Microbiota

Research has made it clear that an imbalance in gut bacteria, often marked by lower microbial diversity and an increase in pathogenic taxa, can produce higher rates of organ rejection, graft dysfunction, and post-transplant mortality. Clinical studies have shown that specific microbial shifts correlate with negative outcomes, including acute rejection and chronic allograft dysfunction, reinforcing the connection between altered microbiota and poorer post-transplant results [16,17,18,19]. These results can be seen in several types of solid organ transplant.

In the setting of kidney transplantation, several studies have found that “microbial distance” can even surpass HLA incompatibility in predicting six-month renal function. Microbiota analyses pre- and post-transplant reveal an increase in Proteobacteria, a pathogenic phylum, and a decrease in the commensal genus Bacteroides [20,21]. This imbalance, characterized by reduced diversity and an elevated presence of pro-inflammatory elements, is associated with organ function deterioration and acute rejection [22]. Long-term evaluations, extending beyond 20 years post-transplant, confirm that there are persistently reduced biodiversity levels [23].

In liver transplantation, recent findings indicate that patients experiencing rejection exhibit significantly lower microbial diversity, as determined by genetic sequencing analyses of fecal samples. These analyses compared pre- and post-transplant microbiota data from patients who experienced rejection with data from those who did not. Reduced microbial diversity may contribute to increased inflammation, raising the risk of rejection [24].

In lung transplant recipients, intestinal dysbiosis, driven by the intensive use of immune suppressants and antibiotics that reduce microbial diversity, has been linked to a heightened incidence of complications, and local bacterial communities, such as those in the lower respiratory tract, have been shown to directly influence acute rejection and graft survival, with lower microbial diversity associated with worse outcomes [25,26].

While immunosuppressive drugs are essential for preventing rejection, they weaken the host’s ability to regulate the bacterial microenvironment, thus exacerbating dysbiosis. In fact, several preclinical studies in murine models have highlighted the immune modulatory role of the intestinal microbiota in rejection mechanisms, regulating T-helper responses and reducing systemic inflammation [23,27].

Collectively, these observations support the hypothesis that preserving a balanced intestinal microbiota is essential to limiting harmful immune activation and improving the prognosis of organ transplantation.

However, the microbiota is not exclusive to the intestine: the ocular surface also harbors a microbial community that interacts with the local immune system. Given that both systems share similar immune mechanisms, it is reasonable to question whether the stability of the ocular surface microbiota might play a comparable role in corneal transplantation. This hypothesis is supported by the immune physiology of the ocular surface, which shares several characteristics with the intestinal immune system, including microbial recognition, mediated by innate immune receptors, and the induction of immune tolerance Fine modulo.

## 6. Parallels Between Gut and Ocular Surface Microbiota in Transplantation

Both the gut and the ocular surface rely on a delicate balance of microbes to maintain immune function and tissue integrity. In these mucosal environments, epithelial and immune cells use toll-like receptors (TLRs) and other innate immune sensors to distinguish between helpful and harmful bacteria. This recognition process determines whether the body launches an inflammatory response—through cytokines like IL-1β, TNF-α, and IL-6—or maintains immune tolerance to beneficial microbes. A healthy microbial community is crucial for keeping epithelial barriers intact. In the gut, enterocytes work alongside the microbiota to prevent harmful pathogens from crossing into the bloodstream. Similarly, the conjunctival and corneal epithelium depend on stable microbial interactions to maintain their protective layers. Additionally, Pathogen-Associated Molecular Patterns (PAMPs) affect the expression of adhesion molecules, cytokines, and chemokines crucial for immune cell recruitment and activation [28,29].

Specifically, PAMPs influence immune reaction through interactions with Pattern Recognition Receptors (PRRs), particularly TLRs, expressed on epithelial and immune cells. Upon PAMP recognition, TLRs trigger intracellular signaling cascades, activating transcription factors such as NF-κB and MAPK. These transcription factors enhance the secretion of chemokines, crucial for recruiting immune cells to sites of inflammation or infection [30]. For instance, LPS from Gram-negative bacteria, recognized by TLR-4, can elevate chemokines like CXCL10, an inflammatory chemokine expressed in both cornea and conjunctiva that is able to mediate immune responses through the activation and recruitment of leukocytes such as T cells, eosinophils, monocytes and NK cells in ocular tissues [31]. Moreover, this activation increases adhesion molecule expression, such as ICAM-1, VCAM-1, and E-selectin, facilitating immune cell adhesion and migration into inflamed areas. In addition, microbial metabolites like short-chain fatty acids (SCFAs)—butyrate, propionate, and acetate—modulate immune responses and reinforce epithelial barrier function. Butyrate, in particular, strongly promotes the expression of regulatory T cells (Tregs) by enhancing histone acetylation at Foxp3 promoter regions, leading to increased Foxp3 expression and strengthened immune regulatory capacity. Tregs are essential for immune homeostasis and the suppression of excessive inflammation [32]. Furthermore, SCFAs enhance barrier function in intestinal epithelial cells (IECs); for instance, germ-free mice colonized with SCFA-producing bacteria such as *Bacteroides thetaiotaomicron* or *Faecalibacterium prausnitziis* show increased goblet cell differentiation and mucus production. Although lacking substantiated ocular studies, these mechanisms suggest potential parallel pathways for ocular surface immunity [33].

The distinction in immune regulation between the gut and the ocular surface reflects the distinct microbial environments of these two anatomical sites. In the gut, the mucosal immune system is continuously exposed to a vast and diverse array of microbiota, necessitating a strong emphasis on immunological tolerance. Here, T-cell subsets—particularly Tregs—are essential in preventing excessive inflammatory responses triggered by commensal microorganisms. Specific commensal bacteria, such as *Bacteroides fragilis* and *Clostridium clusters IV* and *XIVa*, actively support Treg development and function, reinforcing immune tolerance and maintaining intestinal homeostasis.

In contrast, the ocular surface represents a fundamentally different microbial ecosystem, characterized by lower microbial density (“paucibacterial”) and diversity. Consequently, the immune strategy shifts toward more active surveillance and defense against potential pathogens. Here, specific commensal bacteria like *Corynebacterium mastitidis* stimulate T-cell subsets such as γδ T cells and certain T-helper cells to produce cytokines, notably IL-17. Although IL-17 is typically associated with pro-inflammatory responses, in this context it plays a protective role, enhancing the ocular surface’s defense mechanisms against opportunistic pathogens like *Candida albicans* and *Pseudomonas aeruginosa* [3,34].

## 7. Corneal Transplantation: Causes of Graft Failure and the Role of Microbiota

**Figure 1:**
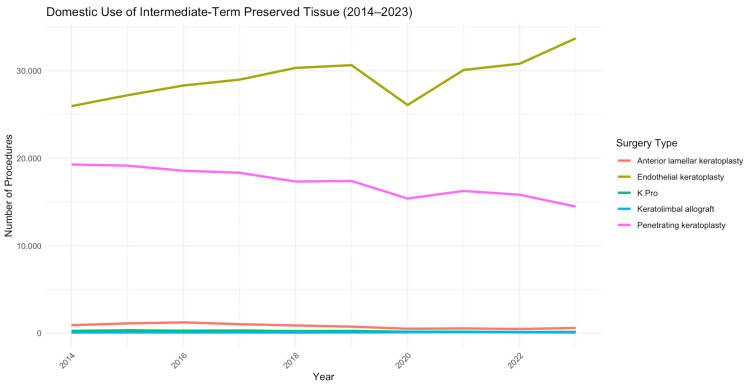
Trends in the number of surgical procedures using preserved ocular tissue in the United States from 2014 to 2023. The curves illustrate temporal variations for different types of surgeries, including penetrating keratoplasty, endothelial keratoplasty, anterior lamellar keratoplasty, keratolimbal allograft, and K-Pro.

The cornea plays a crucial role in vision, and any changes to its shape or clarity can significantly impact eyesight. Corneal opacity (CO) accounts for 3.2% of blindness cases and 1.3% of moderate-to-severe vision impairment, ranking among the top five global causes of blindness [35]. Over the past 30 years, vision loss due to CO has declined, largely due to the increasing success and availability of corneal transplantation [36]. Keratoplasty is the most frequently performed allogeneic transplant worldwide and although transplant numbers dipped in 2020 due to the COVID-19 pandemic, they have since returned to pre-pandemic levels [37]. In the context of corneal transplantation, data from the 2023 Eye Banking Statistical Report indicate that endothelial lamellar transplants are the most frequently performed procedures in the United States, followed by full-thickness corneal transplants and anterior lamellar transplants (Figure 1) [37].

When analyzing the main causes of graft failure in these procedures, primary graft failure is most common in DMEK and DSAEK, resulting from donor, recipient, and surgical factors that cause graft malfunction immediately after surgery, independently of immunological factors or the gradual loss of endothelial cells. Proper donor tissue procurement and storage, as well as the absence of donor–host interface abnormalities, are crucial for successful graft adherence, making immunological rejection a secondary cause of failure in these procedures. Conversely, in penetrating keratoplasty (PK), the second most frequently performed procedure after endothelial lamellar transplantation, the predominant cause of failure is immunological rejection, followed by surface disease, while in DALK (deep anterior lamellar keratoplasty), immunological rejection ranks second after what is broadly referred to as “surface disease”. This term indicates conditions such as limbal stem cell insufficiency, infectious keratitis, keratolysis, or persistent epithelial defect, all of which may compromise transplant success [35,38].

While OSM dysbiosis is unlikely to play a major role in endothelial keratoplasty failure, it may be more relevant in penetrating and anterior lamellar transplants. In these settings, the disruption of the eye’s natural microbial balance can be one of the factors weakening the graft tolerance mechanism, potentially predisposing a patient to rejection.

## 8. Dysbiosis and Corneal Transplantation: A Plausible Connection?

**Figure 2:**
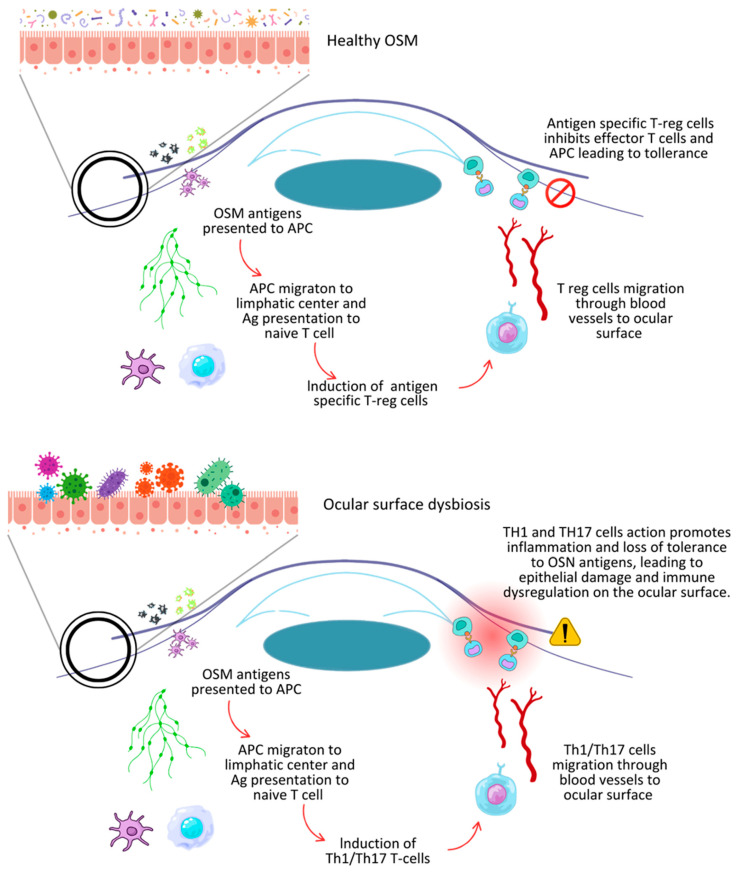
Comparison between healthy ocular surface (**top**) and dysbiosis of ocular surface microbiota (OSM) (**bottom**). In normal conditions, OSM antigen presentation induces expression of Tregs, promoting immune tolerance. In contrast, dysbiosis favors Th1/Th17 cell activation, triggering inflammation, epithelial damage, and immune dysregulation, increasing risk of corneal graft rejection.

The cornea has a unique level of immune privilege, setting it apart from other transplanted organs. Unlike solid organ transplants, the cornea lacks blood and lymphatic vessels, has fewer antigen-presenting cells (APCs), and produces immunosuppressive cytokines like TGF-β and melanocyte-stimulating hormone (MSH). These factors help regulate the immune response and reduce the risk of graft rejection [39]. A key player in maintaining this balance is a mechanism known as ACAID (anterior chamber-associated immune deviation). When antigens enter the anterior chamber of the eye, they are taken up by dendritic cells and macrophages, which then migrate to the cervical lymph nodes. There, they interact with naive T cells, prompting them to differentiate into Tregs [40]. These Tregs can suppress pro-inflammatory responses directed against the same antigen. This delicate balance can be disrupted by neovascularization, which facilitates the transport of antigens and immune cells; by re-transplantation (often associated with lymphangiogenesis); and by inflammation. In such conditions, the expression of MHC class I and II antigens increases, attracting APCs to all layers of the cornea and heightening the risk of rejection [41]. In this context, the dysbiosis of the ocular surface microbiota (OSN), with a resulting shift toward a pro-inflammatory state, can disrupt the fragile immunological equilibrium that ensures tolerance, fueling immune system changes and increasing the likelihood of graft rejection (Figure 2).

## 9. Ocular Surface Diseases and Their Link to Microbial Dysbiosis

Regarding a secondary factor in corneal transplant failure, ocular surface diseases, published evidence has already documented the very substantial relationship with dysbiotic ocular surface and conditions such as dry eye disease (DED), microbial keratitis, and chronic allergic conjunctivitis.

In DED, microbial dysbiosis can disrupt immune tolerance to the eye’s natural microbiota, leading to chronic inflammation, tissue damage, and tear film instability—all key hallmarks of the disease. Studies have shown that individuals with DED have distinct microbial changes compared to healthy individuals. In fact, the conjunctival microbiota of DED patients exhibits lower alpha diversity, indicating a reduced variety of bacterial species, which may contribute to inflammation and disease progression [42,43].

Several studies have also linked conjunctival microbiota imbalances to allergic conjunctivitis, particularly in vernal keratoconjunctivitis (VKC), a severe and chronic form of the disease. There are increased relative abundances of *Moraxella*, *Staphylococcus*, *Streptococcus*, and *Corynebacterium* in VKC patients compared to healthy controls, alongside decreased levels of Acinetobacter and Pseudomonas. These microbial shifts may worsen inflammation, alter immune responses, and interact with host immune pathways, contributing to VKC pathophysiology [44,45].

Also, extensive clinical and experimental evidence has established a link between ocular microbiota dysbiosis and bacterial keratitis. Shivaji et al. analyzed conjunctival bacterial microbiomes in healthy individuals and patients with bacterial keratitis, revealing significant differences in alpha diversity indices, bacterial phyla and genera composition, and functional analyses. These findings demonstrated a clear distinction between the microbiomes of healthy controls and bacterial keratitis patients [46].

## 10. The Ocular Surface Microbiota in Keratoconus: Emerging Insights

A growing area of research is the connection between the OSM and diseases that lead to corneal transplantation, particularly keratoconus. Traditionally considered a structural disorder, emerging evidence suggests that microbial imbalances may also play a role in keratoconus pathogenesis. In fact, OSM dysbiosis can potentially influence corneal stability and disease progression. Recent studies have found notable differences in the microbiota of keratoconus patients compared to healthy individuals. Specifically, the bacterial genera *Pelomonas* and *Ralstonia* were only detected in keratoconus patients, while *Corynebacterium* and *Kocuria* were exclusive to healthy controls. This distinct microbial pattern suggests that specific bacterial communities may contribute to keratoconus development, either by promoting inflammation or disrupting corneal homeostasis. Additionally, patients with keratoconus show reduced microbial diversity, as indicated by the lower Shannon indices compared to controls. This reduced microbial diversity compromises the resilience of the OSM to imbalances, potentially promoting pathological processes involving chronic inflammation and corneal tissue remodeling [47].

## 11. Current Knowledge Gaps

Our group reviewed the current PubMed and Scopus published literature using the following search strings:

(“ocular surface microbiome” OR “ocular microbiota” OR “conjunctival microbiome”) AND (“corneal transplant” OR keratoplasty) AND (outcome OR prognosis OR “treatment outcome” OR success OR failure);

(“ocular surface microbiome” OR “ocular microbiome” OR “conjunctival microbiome” OR “ocular flora” OR “eye microbiome” OR “ocular microbiota”) AND (“corneal transplantation” OR “corneal graft” OR keratoplasty) AND (complications OR infection OR “graft rejection” OR “graft failure” OR “graft survival”).

Our detailed analysis of the current literature, conducted using the previously outlined criteria, did not identify any studies explicitly investigating the direct role of ocular surface microbiota in corneal transplantation outcomes. It should be noted that this absence of results might be partially influenced by the limited combination of keywords or by the specificity of our search terms, which intentionally focused on direct links between the ocular microbiome and corneal transplantation outcomes. While associations between ocular surface microbiota dysbiosis and related ocular diseases have been documented, such studies were intentionally excluded from this review, as our primary objective was to identify direct evidence specifically linking microbiome alterations to corneal graft success or failure. The absence of such studies represents a notable knowledge gap, emphasizing the importance of future targeted research in this area.

## 12. Conclusions and Future Perspectives

The growing understanding of the microbiota’s influence on immune regulation in solid organ transplantation raises fundamental questions about the potential role of the ocular surface microbiota (OSM) in the success of corneal transplantation. Although evidence in this field remains limited, the parallels between the immune mechanisms of the intestinal mucosa and the ocular surface suggest that microbial homeostasis may play a role in graft tolerance.

The lack of dedicated studies on the relationship between OSM and corneal transplantation represents a significant gap in the scientific literature. Future research should prioritize prospective longitudinal studies examining microbiota composition before and after keratoplasty to elucidate correlations with graft survival, rejection rates, and other postoperative complications. Systematic reviews and meta-analyses would also be valuable for periodically summarizing evolving data, thereby guiding evidence-based clinical practices. Moreover, developing standardized methodologies for ocular microbiome analysis through advancements in metagenomics and bioinformatics would facilitate comparability among studies and enhance our understanding of ocular microbial dynamics.

A deeper understanding of ocular microbiota may enable the exploration of targeted therapeutic interventions, including ocular probiotics, postbiotics, or microbiome-modulating treatments. Promising examples of these strategies already exist, such as a patented formulation using a fermented postbiotic derived from *Lactobacillus paracasei*. Unlike traditional probiotics, this postbiotic avoids introducing living microorganisms or PAMPs such as lipopolysaccharide (LPS) and lipoteichoic acid (LTA), thereby minimizing potential ocular irritation and inflammation [48]. This innovative approach has demonstrated the potential to modulate ocular inflammation, promote Tregs, and enhance epithelial barrier function, thus offering a therapeutic avenue without the adverse effects typically associated with long-term topical treatments. Ultimately, addressing these research gaps could lead to personalized therapeutic strategies tailored to individual microbiome profiles, significantly optimizing clinical management and patient care in corneal transplantation.
